# Psychosocial assessment in liver transplantation (LT): an analysis of short-term outcomes

**DOI:** 10.1097/HC9.0000000000000017

**Published:** 2023-01-10

**Authors:** Lindsay A. Matthews, Jessica A. Musto, Nimrod Deiss-Yehiely, Kimberly E. Daniel, Christina Lightbourn, Maureen Garvey, Fay Osman, David P. Foley, John R. Rice, Michael R. Lucey

**Affiliations:** 1 Department of Medicine, University of Wisconsin-Madison, Madison, Wisconsin, USA; 2 Division of Gastroenterology & Hepatology, University of Wisconsin-Madison, Madison, Wisconsin, USA; 3 Department of Surgery, University of Wisconsin-Madison, Madison, Wisconsin, USA

## Abstract

Our research showed that patients with alcohol-associated liver disease (ALD) had more severe liver disease than those without a diagnosis of ALD yet were less likely to be selected for transplant listing due to their increased psychosocial vulnerability. This study aims to answer whether this vulnerability translates to worse short-term outcomes after transplant listing.

**Methods:**

A total of 187 patients were approved for liver transplant listing and are included in the present retrospective study. We collected dates of transplantation, retransplantation, death, and pathologic data for evidence of rejection, and reviewed alcohol biomarkers and documentation for evidence of alcohol use.

**Results:**

The ALD cohort had higher Stanford Integrated Psychosocial Assessment for Transplant (SIPAT) scores (39.4 vs. 22.5, *p* <0.001) and Model for End-Stage Liver Disease (MELD)-Na scores (25.0 vs. 18.5, *p* <0.001) compared with the non-ALD cohort. Forty-nine (59.7%) subjects with ALD and 60 (57.1%, *p* =0.71) subjects without ALD subsequently received a liver transplant. Overall mortality was similar between the 2 groups (20.7% ALD vs. 21.0% non-ALD, *p* =0.97). Neither the SIPAT score (HR: 0.98, 95% CI: 0.96–1.00, *p* =0.11) nor MELD-Na score (HR 0.99, 95% CI 0.95-1.02, *p* =0.40) were associated with mortality. Patients with ALD were more likely to have alcohol biomarkers tested both before (84.1% vs. 24.8% non-ALD, *p* <0.001) and after liver transplantation (74.0% vs. 16.7% non-ALD, *p* <0.001). SIPAT score was associated with alcohol use after listing (OR: 1.03, 95% CI: 1.0–1.07, *p* =0.04), although a return to alcohol use was not associated with mortality (HR: 1.60, 95% CI: 0.63–4.10, *p* =0.33).

**Conclusion:**

Patients with ALD had higher psychosocial risk compared with patients without a diagnosis of ALD who were placed on the waitlist, but had similar short-term outcomes including mortality, transplantation, and rejection. Although a high SIPAT score was predictive of alcohol use, in the short-term, alcohol use after transplant listing was not associated with mortality.

## INTRODUCTION

Alcohol-associated liver disease (ALD) is now the most common indication for liver transplantation (LT) in the US, accounting for nearly 40% of liver transplants annually.^1^ Its prevalence is expected to increase in the coming years, in part from increased alcohol consumption during the COVID-19 pandemic.^2^ Almost invariably, the ALD population has comorbid alcohol use disorder (AUD) and there is concern that selecting patients with AUD may jeopardize the allograft due to a relapse to harmful alcohol use.^3^ This has led to an ongoing debate on how best to select patients to minimize the risk of alcohol relapse after LT.

A key component of the selection process is the psychosocial assessment, which generally focuses on a candidate’s social support, substance use disorder history, psychiatric illness, and behavioral problems. This evaluation can be contentious, and committee members cite associated feelings of unfairness and subjectivity.^4^ Standardized tools have been developed to promote equity, fairness, and transparency between patients and transplant centers, and help create a more holistic assessment of risk rather than relying on a fixed period of abstinence, such as the “6-month rule” commonly used in the past. The work of DiMartini et al.^5^ has shown that alcohol use can occur years after LT even after initial sobriety, highlighting the need for long-term monitoring. If recognized, this presents an opportunity to intervene and refer the patient for further medical treatment.

Recently, we published a retrospective assessment of selection for LT at our center in which we used the Stanford Integrated Psychosocial Assessment for Transplant (SIPAT).^6^ SIPAT is a multifaceted, semi-structured tool derived for psychosocial evaluation in solid organ transplant, in which higher scores indicate greater psychosocial vulnerability. Other groups have shown that higher SIPAT scores are associated with immunosuppression nonadherence and allograft rejection.^7^ Despite this, at present, there is no predetermined score above which listing is prohibited. Rather, the SIPAT score is used as a signpost to signify higher vulnerability and the potential need for increased support.

We demonstrated^6^ that that psychosocial assessment had a greater influence on the selection for LT listing in patients with ALD than the urgency of liver failure, whereas it had a relatively minor influence on the non-ALD candidates. Patients with ALD had higher Model for End-Stage Liver Disease (MELD)-Na and SIPAT scores compared with patients without ALD. However, despite their more severe liver disease, patients with ALD were less likely than patients without ALD to be selected for transplant listing. Furthermore, although the patients with ALD selected for listing had lower (ie, more favorable) SIPAT scores than those with ALD who were declined listing, the SIPAT scores in the selected patients with ALD were still significantly higher than those without ALD. We concluded that the ALD population presenting for evaluation had profound psychosocial impediments. While we tend to select the least at-risk patients with ALD, those selected for LT listing still carried a significantly greater risk of future relapse into harmful drinking, allograft injury, and allograft loss than patients without ALD selected during the same period.

However, our previous study did not address whether the greater assessed vulnerability of the selected patients with ALD translated to worse outcomes compared with their peers without ALD. We aim to answer this question in the present paper, at least in the short-term encompassing the first 2–3 years after transplant listing. We examine significant outcomes between the ALD and non-ALD groups such as transplantation, graft and patient survival, and consumption of alcohol, and their association with the psychosocial assessment, using the SIPAT score as our psychosocial “assay.”

## METHODS

### Study population

The 187 patients in our cohort were presented and approved for transplant listing at our center’s multidisciplinary liver transplant selection committee between June 2018 and December 2019. Our follow-up period ended in October 2021. Patients were grouped by liver disease etiology as either ALD or non-ALD (Table 1). MELD-Na and SIPAT scores^9^ at the time of presentation to the transplantation selection committee were recorded.^6^ The electronic medical record (EMR) was surveyed and where a formal assessment of AUD by an addiction medicine counselor was extant, the severity of AUD was recorded. All research was conducted in accordance with both the Declarations of Helsinki and Istanbul. All research was approved by the University of Wisconsin Institutional Review Board. Informed consent was waived by the IRB.

**TABLE 1:** Cohort characteristics

	ALD (n=82)	Non-ALD (n=105)	*p*
Age, y (SD)	52.3 (11.1)	55.9 (10.7)	0.03^a^
Male, n (%)	54 (65.9)	61 (58.1)	0.28
White race, n (%)	77 (95.1)	98 (93.3)	0.69
Global SIPAT, mean (SD)	39.4 (15.1)	22.5 (12.4)	<0.001^a^
MELD-Na, mean (SD)	25.0 (10.1)	18.5 (8.2)	<0.001^a^
Rejection, n (%)	17 (20.7)	12 (11.4)	0.08
Transplanted, n (%)	49 (59.7)	60 (57.1)	0.71
Retransplantation, n (%)	4 (4.9)	2 (1.9)	0.49
Death, n (%)	17 (20.7)	22 (21.0)	0.97
Years follow-up, mean (SD)	2.49 (0.59)	2.50 (0.52)	0.97
Return to alcohol use, n (%)	10 (12.2)	6 (5.7)	0.12
Biomarkers checked pre-LT, n (%)	69 (84.1)	26 (24.8)	<0.001^a^
Biomarkers checked post-LT, n (%)	37 (74.0)	10 (16.7)	<0.001^a^
Alcohol use disorder, severity^8^			
No grade, n (%)	9 (11.0)		
Mild, n (%)	5 (6.1)		
Moderate, n (%)	18 (22.0)		
Severe, n (%)	50 (61.0)		
Alcohol use disorder			
* *In remission >6 mo^b^, n (%)	36 (43.9)		
Active or <6 mo remission^b^, n (%)	46 (56.1)		
Psychiatric treatment before LT			
* *None, n (%)	33 (40.2)		
Pharmacotherapy, n (%)	3 (3.7)		
Psychotherapy, n (%)	39 (47.6)		
* *Pharmacotherapy+psychotherapy, n (%)	7 (8.5)		
Etiology of liver disease^c^			
ALD, n (%)	67 (35.8)		
AAH, n (%)	12 (6.4)		
ALD+HCV, n (%)	3 (1.6)		
HCV, n (%)		2 (1.1)	
NASH, n (%)		38 (20.3)	
HCC, n (%)		12 (6.4)	
* *PBC+PSC+AIH, n (%)		32 (17.1)	
Other, n (%)		21 (11.2)	

^a^ *p*-value < 0.05 was considered statistically significant.

^b^ From date of committee evaluation.

^c^ Percentages of overall cohort (n=187).

Abbreviations: AAH indicates acute alcohol-associated hepatitis; AIH, autoimmune hepatitis; ALD, alcohol-associated liver disease; LT, liver transplantation; PBC, primary biliary cirrhosis; PSC, primary sclerosing cholangitis.

### Transplantation outcomes

The EMR of each subject was reviewed to record key dates including, where appropriate, removal from waiting list, transplantation, retransplantation, death, and the most recent direct contact within our health system. Whenever a liver biopsy was performed post-transplant, the pathologic data were reviewed for evidence of allograft rejection.

### Alcohol use

We reviewed the EMR for laboratory data on biomarkers of alcohol use, including serum ethanol, urinary ethylglucuronide, and whole blood phosphatidylethanol (PEth).^10^ Discrete episodes of testing for alcohol biomarkers were counted and categorized as pre-transplant or post-transplant. Testing was performed at the discretion of the managing physician. In addition to alcohol biomarkers, hepatology clinic notes and inpatient hepatology consult notes were reviewed to determine if the subject self-reported any return to alcohol use. The subject was identified as returning to “any alcohol use” with the presence of any positive metabolite or disclosure of alcohol use to their provider. In addition, we characterized “harmful drinking” as >14 drinks per week for men or >7 for women,^11^ or by any whole blood PEth level >112 ng/mL if the subject did not disclose their quantity of alcohol consumption.^12^

### Statistical methodology

All baseline study characteristics were summarized using counts and frequencies. Categorical outcomes were compared using chi-squared tests and Fisher exact tests for cells <5. Continuous variables were summarized using mean plus SD and made comparisons using a student *t* test or the Wilcoxon sign rank test for data that are not normally distributed. For patient and graft survival, a Cox proportional hazard model was used to predict risk factors for mortality or retransplants. We compared the equality of survival functions for significance using log-rank tests and life tables. A Logistical regression model was constructed to determine factors associated with rejection. We constructed survival curves using the Kaplan-Meier method and the Nelson Aalen cumulative hazard curves. All *p-*values if ≤0.05 were considered statistically significant. We used the STATA survival analysis package in version 17 to conduct all analyses (StataCorp. 2021. *Stata Statistical Software: Release 17*. College Station, TX: StataCorp LLC).

## RESULTS

### Baseline characteristics

The cohort (Table 1) comprised 187 individuals, 82 with ALD and 105 with a non-ALD diagnosis. The etiology of liver disease is presented in Table 1. The cohort was disproportionately male (65.9% ALD, 58.1% non-ALD, *p* =0.28) and almost exclusively of White race (95.1% ALD, 93.3% non-ALD, *p* =0.69). As compared with the non-ALD cohort, the ALD cohort had both higher global SIPAT scores (39.4 vs. 22.5, *p* <0.001) and MELD-Na scores at the time of committee review (25.0 vs. 18.5, *p* <0.001). Of the ALD cohort, 43.9% had AUD in remission for >6 months before committee evaluation, and 61.0% had severe AUD. Before transplant, 39 (47.6%) patients with ALD underwent psychotherapy, 3 (3.7%) started psychotropic medications, and 7 (8.5%) started both psychotherapy and psychotropic medications.

### Transplantation outcomes

**FIGURE 1:**
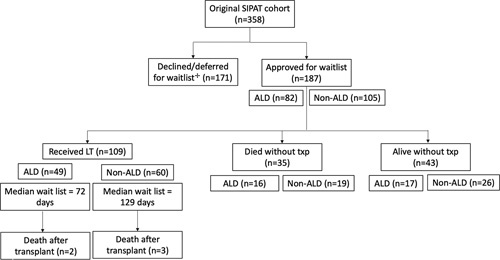
Subject flowchart. ^+^See Daniel et al.^6^ for more details. Abbreviations: ALD indicates alcohol-associated liver disease; LT, liver transplantation; SIPAT, Stanford Integrated Psychosocial Assessment for Transplant.

At the close of the data collection interval, 49 (59.7%) ALD subjects and 60 (57.1%, *p* =0.60) non-ALD subjects had received a liver transplant, 16 (18.3%) ALD and 19 (17.8%) non-ALD subjects had died without a transplant, and 17 (20.7%) ALD and 26 (24.8%) non-ALD subjects were alive without having received a transplant. Two subjects with ALD and 3 with non-ALD died after transplantation. The median interval on the waiting list among patients who received transplants was 72 days for ALD and 129 days for non-ALD (*p* =0.17) (Figure 1).

During a follow-up period of about 30 months (mean: 2.49 y ALD vs. 2.50 y non-ALD, *p* =0.97), mortality after listing was similar between the 2 groups (20.7% ALD vs. 21.0% non-ALD, *p* =0.97). There was a trend towards higher rates of biopsy-proven rejection in the ALD group (20.7% ALD vs. 11.4% non-ALD, *p* =0.08) (Table 1). On univariable analysis (Table 2), transplantation was associated with decreased mortality (HR: 0.1, 95% CI: 0.03–0.20, *p* <0.001). Neither the SIPAT score (HR: 0.98, 95% CI: 0.96–1.00, *p* =0.11) nor the MELD-Na score at the time of committee review (HR: 0.99, 95% CI: 0.95–1.02, *p* =0.40) were associated with mortality.

**TABLE 2:** Predictors of mortality in overall cohort (n=187)

	Univariable analysis			Multivariable analysis^b^		
	HR	95% CI	*p*	HR	95% CI	*p*
ALD diagnosis	1.02	0.54–1.92	0.95	1.47	0.72–3.01	0.29
SIPAT score	0.98	0.96–1.00	0.11	0.98	0.95–1.0	0.11
MELD-Na	0.99	0.95–1.02	0.40	0.99	0.96–1.03	0.97
Transplantation	0.10	0.03–0.20	<0.001^a^			
Return to alcohol use	1.60	0.63–4.10	0.33			

^a^ *p*-value < 0.05 was considered statistically significant.

^b^ Adjusted for MELD-Na, SIPAT score, age, sex, diagnosis (ALD vs. non-ALD)

Abbreviations: ALD indicates alcohol-associated liver disease; MELD, Model for End-Stage Liver Disease; SIPAT, Stanford Integrated Psychosocial Assessment for Transplant.

Among transplanted patients (Table 3), SIPAT and MELD-Na scores were predictive of rejection, although this was not seen in multivariable analysis. There was a trend towards rejection in patients with ALD (HR: 1.97, 95% CI: 0.92–4.21, *p* =0.08) compared with patients without ALD.

**TABLE 3:** Predictors of rejection in transplanted patients (n=109)

	Univariable analysis			Multivariable analysis^b^		
	HR	95% CI	*p*	HR	95% CI	*p*
ALD diagnosis	1.97	0.92–4.21	0.08	1.27	0.48–3.32	0.63
SIPAT score	1.02	1.0–1.04	0.04^a^	0.99	0.96–1.02	0.59
MELD-Na	1.04	1.0–1.08	0.05^a^	1.01	0.97–1.06	0.56
Return to alcohol use	0.46	0.1–3.35	0.44			

^a^ *p*-value < 0.05 was considered statistically significant.

^b^ Adjusted for MELD-Na, SIPAT score, age, sex, diagnosis (ALD vs. non-ALD).

Abbreviations: ALD indicates alcohol-associated liver disease; MELD, Model for End-Stage Liver Disease; SIPAT, Stanford Integrated Psychosocial Assessment for Transplant.

### Alcohol use

**FIGURE 2:**
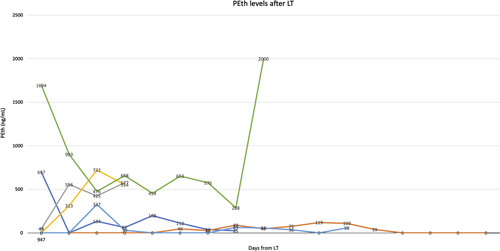
PEth levels of patients with at least 1 positive test result (n=7). Abbreviations: LT indicates liver transplantation; Peth, phosphatidylethanol.

Patients with ALD were more likely to have alcohol biomarkers tested both before (84.1% vs. 24.8% non-ALD, *p* <0.001) and after LT (74.0% vs. 16.7% non-ALD, *p* <0.001) (Table 1). Alcohol use after transplant listing was uncommon, and there was no significant difference between the 2 groups: 10 ALD subjects (12.2%) versus 6 (5.7%) non-ALD subjects (*p* =0.12). The quantitative PEth results of the 7 patients in whom there was a detectable level after transplant are presented in Figure 2. Eight subjects in the ALD group returned to harmful drinking after LT listing, whereas no one in the non-ALD group displayed harmful alcohol use. Return to alcohol use after listing was not associated with mortality (HR: 1.60, 95% CI: 0.63–4.10, *p* =0.33). Among transplanted patients (Table 3), a return to alcohol use was not associated with rejection (HR: 0.46, CI: 0.1–3.35, *p* =0.44).

On univariable analysis (Table 4), global SIPAT score was associated with alcohol use (OR: 1.03, 95% CI: 1.0–1.07, *p* =0.04). Subscores for readiness (OR: 1.31, 95% CI: 1.09–1.57, *p* =0.004), psychosocial comorbidities (OR: 1.11, 95% CI: 1.02–1.20, *p* =0.02), and substance use (OR: 1.14, 95% CI: 1.04–1.26, *p* =0.006) were associated with alcohol use during the interval of observation, whereas the subscore for social support (OR: 1.07, 95% CI: 0.89–1.28, *p* =0.50) was not associated with a return to alcohol use.

**TABLE 4:** Predictors of a return to alcohol use in overall cohort (n=187)

	Univariable analysis			Multivariable analysis^b^		
	OR	95% CI	*p*	OR	95% CI	*p*
ALD	2.29	0.80–6.59	0.12	1.25	0.35–4.45	0.73
Age (y)	0.98	0.93–1.02	0.29	0.99	0.94–1.04	0.70
MELD-Na	1.04	0.99–1.10	0.13	1.02	0.96–1.08	0.48
Global SIPAT score	1.03	1.0–1.07	0.04^a^	1.02	0.98–1.06	0.26
* *Readiness	1.31	1.09–1.57	0.004^a^			
* *Psychopathology	1.11	1.02–1.20	0.02^a^			
* *Substance use	1.14	1.04–1.26	0.006^a^			
* *Social support	1.07	0.89–1.28	0.50			

^a^ *p*-value < 0.05 was considered statistically significant.

^b^ Adjusted for MELD-Na, SIPAT score, age, sex, diagnosis (ALD vs. non-ALD).

Abbreviations: ALD indicates alcohol-associated liver disease; MELD, Model for End-Stage Liver Disease; SIPAT, Stanford Integrated Psychosocial Assessment for Transplant.

## DISCUSSION

### Transplantation outcomes

**FIGURE 3:**
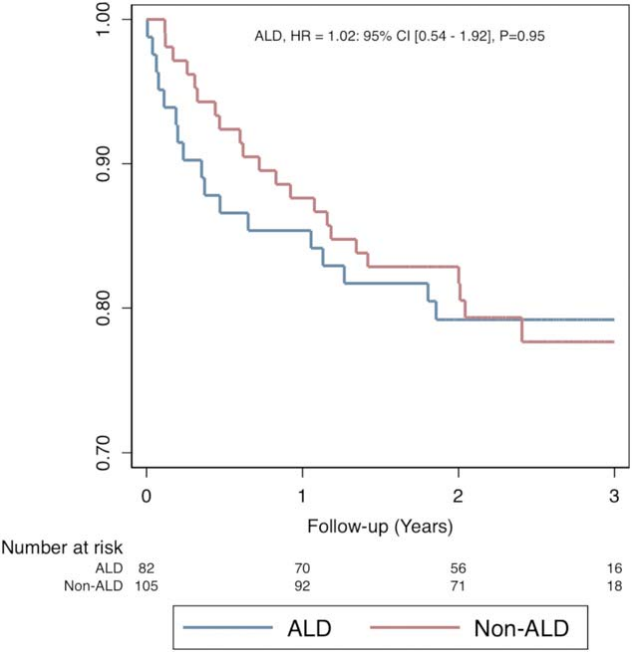
Patient survival in overall cohort. Date of committee evaluation is time 0. Abbreviations: ALD indicates alcohol-associated liver disease.

Rates of transplantation were similar between the ALD and non-ALD groups. The ALD group had a shorter median time on the waitlist, likely due to their higher MELD-Na scores. We interpret this to show that in contrast to the significantly lower rate of selection of patients with ALD in our cohort, once placed on the waiting list, patients proceed to transplant irrespective of diagnosis.^6^ Mortality (Figure 3) and rejection were similar in the ALD and non-ALD groups, showing that even very ill patients with ALD can achieve satisfactory short-term results. Among those transplanted, SIPAT and MELD-Na scores were associated with rejection, although this association was not seen in multivariable analysis. Notably, most episodes of acute cellular rejection occur within a year of transplant and are thus included in our data. However, our study is unable to consider either late-onset T-cell–mediated or vasculopathic chronic rejection after transplantation.^13^,^14^ Our cohort of patients with ALD was nearly split between patients with ongoing AUD and those who had sustained remission for >6 months before committee evaluation. Most patients had severe AUD. Although the ALD group had a shorter median time on the waitlist, the majority engaged with mental health professionals before transplant with the administration of psychotherapy, pharmacotherapy, or both. This utilization of therapy for AUD contrasts with the experience of most patients with ALD who are rarely given professionally administered treatment for AUD.^15^

### Return to alcohol use

Higher SIPAT scores, indicating more psychosocial vulnerability at the time of listing, correlated with a return to alcohol use after listing. However, alcohol use after transplant was uncommon and was seen in both ALD and non-ALD groups. While alcohol use did not correlate with death or graft loss, both rare events, we would counsel against the notion that our data show that alcohol use was harmless. Although there was no statistically significant difference in the rate of return to any alcohol use after LT listing by ALD and non-ALD recipients alike, it is important to note that nearly all subjects in the ALD group who returned to alcohol use had evidence of harmful drinking, compared with the absence of harmful drinking by non-ALD subjects. Several patients denied alcohol use before discovery of high PEth levels, highlighting the importance of biomarkers in detecting alcohol use, which can promote discussions surrounding use and referrals for increased support. Notably, nearly all patients received a blood transfusion in the peritransplant period, which can cause false-positive results on PEth testing from alcohol use in the blood donor pool.^16^ Although this caveat has important implications in the immediate postoperative period, it is less of a concern longitudinally during periods of clinical stability. Our previous studies and those of other groups have shown that harmful drinking post-LT can lead to liver damage, fibrosis, and eventual graft loss, the latter of which can occur after years of use and may not be accounted for in our follow-up period.^17^ Our data suggest that this risk is confined to the ALD population.

### Alcohol biomarkers

As we expected, our data show that alcohol biomarkers were ordered more frequently in patients with ALD. However, we were surprised to discover that we were not consistent in applying biomarker testing or interview techniques to detect alcohol use. Our institution does not have a set policy on obtaining biomarker testing or discussing alcohol use at follow-up, leaving this to the discretion of each managing physician. PEth was tested more frequently post-transplant compared with other biomarkers, often as a standalone test. It is important to screen for alcohol use in all patients, as alcohol use is associated with decreased graft survival.^17^ Recognizing a return to alcohol use presents an opportunity for early intervention, including counseling and referral to addiction medicine specialists, with the goal of preventing a “slip” from progressing to harmful and chronic use. If alcohol screening is not routine, patients may be hesitant to disclose their use to their provider, fearing that doing so could increase further scrutiny, disapproval, or limitation of care.^18^

### Study limitations

Our study was limited by its relatively short-term follow-up period and retrospective nature. In addition, we likely underestimated alcohol use due to the incomplete application of alcohol biomarker tests postlisting and the reluctance of patients to openly discuss use with their providers. There were no set policies for ordering alcohol biomarkers or discussing alcohol use at follow-up, nor was there a defined process for referral or treatment once alcohol use was identified.

### Future directions

So where do these data lead us in answering the questions regarding the appropriateness of psychosocial assessment for LT? Clearly, the psychosocial assessment in current use causes patients with ALD to undergo a more stringent evaluation, which excludes many from being listed for LT. The consequences of this exclusion for patients with high MELD-Na scores are grim, with up two-thirds dying within 90 days.^19^ The picture is complicated by AUD, since some of the attrition is a result of alcohol relapse; it remains possible that some of the excluded patients could have survived and recovered from liver failure had they received an LT. The present study confirms that a minority of the patients with ALD selected for LT will return to harmful drinking in the short term after LT, however, this risk remains elevated for years after LT.^5^ We plan to continue to follow our cohort to further examine alcohol use in the medium term and long term. We will adopt universal and frequent alcohol biomarker testing in all potential liver transplant recipients as new biomarker testing increases the sensitivity of detecting alcohol use, including serum PEth which remains positive for up to 28 days after alcohol use.^20^ Clear, written policies about alcohol screening promote fairness and equity among transplant recipients, and removes biases associated with only checking those the clinician subjectively believes are high risk. These policies would also equalize scrutiny between different providers and transplant centers and work towards promoting a more equitable and fair transplantation process, which will be of the utmost importance as ALD rates continue to rise.

## CONCLUSIONS

- Patients with ALD had similar outcomes including mortality, transplantation, and rejection as patients without ALD, despite their increased vulnerability as evidenced by higher MELD-Na and SIPAT scores.
- Returning to alcohol use was uncommon; however, a high SIPAT score was predictive. In the short-term, this was not associated with mortality.
- Patients in both the ALD and non-ALD groups returned to alcohol use after LT, although patients with ALD more frequently returned to harmful drinking patterns.
- Alcohol biomarkers were more likely to be checked in patients with ALD, although were checked inconsistently.
- Universal and frequent biomarker testing promotes fairness and equity among transplant recipients and those who remain on the waitlist, and detection of alcohol use presents an opportunity for increased support.

## References

[1] Lee BP Vittinghoff E Dodge JL Cullaro G Terrault NA. National trends and long-term outcomes of liver transplant for alcohol-associated liver disease in the United States. JAMA Intern Med. 2019;179:340–348. 30667468 10.1001/jamainternmed.2018.6536 PMC6439700

[2] Julien J Ayer T Tapper EB Barbosa C Dowd WN Chhatwal J. Effect of increased alcohol consumption during COVID-19 pandemic on alcohol-associated liver disease: a modeling study. Hepatology. 2022;75:1480–1490. 34878683 10.1002/hep.32272 PMC9015640

[3] Mathurin P Lucey MR. Alcohol, liver disease, and transplantation: shifting attitudes and new understanding leads to changes in practice. Curr Opin Organ Transplant. 2018;23:175–179. 29394165 10.1097/MOT.0000000000000517

[4] Volk ML Biggins SW Huang MA Argo CK Fontana RJ Anspach RR. Decision making in liver transplant selection committees: a multicenter study. Ann Intern Med. 2011;155:503–508. 22007044 10.1059/0003-4819-155-8-201110180-00006 PMC3197782

[5] DiMartini A Dew MA Day N. Trajectories of alcohol consumption following liver transplantation. Am J Transplant. 2010;10:2305–2312. 20726963 10.1111/j.1600-6143.2010.03232.x PMC3040647

[6] Daniel KE Matthews LA Deiss-Yehiely N Myers J Garvey M Rice JP. Psychosocial assessment rather than severity of liver failure dominates selection for liver transplantation in patients with alcohol-related liver disease. Liver Transpl. 2022;28:936–944. 34596955 10.1002/lt.26324

[7] Deutsch-Link S Weinberg EM Bittermann T McDougal M Dhariwal A Jones LS. The Stanford Integrated Psychosocial Assessment for Transplant is associated with outcomes before and after liver transplantation. Liver Transpl. 2021;27:652–667. 33320417 10.1002/lt.25975 PMC9531321

[8] American Psychiatric Association. Substance-related and addictive disorders. Diagnostic and Statistical Manual of Mental Disorders, 5th ed. Washington, DC: American Psychiatric Association; 2013.

[9] Maldonado JR Dubois HC David EE Sher Y Lolak S Dyal J. The Stanford Integrated Psychosocial Assessment for Transplantation (SIPAT): a new tool for the psychosocial evaluation of pre-transplant candidates. Psychosomatics. 2012;53:123–132. 22424160 10.1016/j.psym.2011.12.012

[10] Cabezas J Lucey MR Bataller R. Biomarkers for monitoring alcohol use. Clin Liver Dis (Hoboken). 2016;8:59–63. 31041064 10.1002/cld.571 PMC6490197

[11] US Preventive Services Task Force. Screening and behavioral counseling interventions in primary care to reduce alcohol misuse: recommendation statement. Ann Intern Med. 2004;140:554–556. 15068984 10.7326/0003-4819-140-7-200404060-00016

[12] Schröck A Wurst FM Thon N Weinmann W. Assessing phosphatidylethanol (PEth) levels reflecting different drinking habits in comparison to the alcohol use disorders identification test—C (AUDIT-C). Drug Alcohol Depend. 2017;178:80–86. 28645063 10.1016/j.drugalcdep.2017.04.026

[13] Choudhary NS Saigal S Bansal RK Saraf N Gautam D Soin AS. Acute and chronic rejection after liver transplantation: what a clinician needs to know. J Clin Exp Hepatol. 2017;7:358–366. 29234201 10.1016/j.jceh.2017.10.003 PMC5715482

[14] Levitsky J. Acute rejection increases risk of graft failure and death in recent liver transplant recipients. Clin Gastroenterol Hepatol. 2017;15:584–593 e2. 27567694 10.1016/j.cgh.2016.07.035 PMC5326609

[15] Rogal S. Impact of alcohol use disorder treatment on clinical outcomes among patients with cirrhosis. Hepatology. 2020;71:2080–2092. 31758811 10.1002/hep.31042 PMC8032461

[16] Bhavsar-Burke I Guardiola JJ Holden JH. Is blood transfusion a means of false positive phosphatidylethanol testing? Liver Transpl. 2022;28:138–139. 34519152 10.1002/lt.26297

[17] Rice JP Eickhoff J Agni R Ghufran A Brahmbhatt R Lucey MR. Abusive drinking after liver transplantation is associated with allograft loss and advanced allograft fibrosis. Liver Transpl. 2013;19:1377–1386. 24115392 10.1002/lt.23762

[18] Weinrieb RM. Interpreting the significance of drinking by alcohol-dependent liver transplant patients: fostering candor is the key to recovery. Liver Transpl. 2000;6:769–776. 11084066 10.1053/jlts.2000.18497

[19] Musto J Stanfield D Ley D Lucey MR Eickhoff J Rice JP. Recovery and outcomes of patients denied early liver transplantation for severe alcohol-associated hepatitis. Hepatology. 2022;75:104–114. 34387875 10.1002/hep.32110

[20] Hansson P Caron M Johnson G Gustavsson L Alling C. Blood phosphatidylethanol as a marker of alcohol abuse: levels in alcoholic males during withdrawal. Alcohol Clin Exp Res. 1997;21:108–110. 9046381

